# Effect of Agricultural Amendments on *Cajanus cajan* (Pigeon Pea) and Its Rhizospheric Microbial Communities – A Comparison between Chemical Fertilizers and Bioinoculants

**DOI:** 10.1371/journal.pone.0132770

**Published:** 2015-07-31

**Authors:** Rashi Gupta, V. S. Bisaria, Shilpi Sharma

**Affiliations:** Department of Biochemical Engineering and Biotechnology, Indian Institute of Technology Delhi, Hauz Khas, New Delhi 110016, India; Graz University of Technology (TU Graz), AUSTRIA

## Abstract

Inoculation of leguminous seeds with bioinoculants has been practiced in agriculture for decades to ameliorate grain yield by enhanced growth parameters and soil fertility. However, effective enhancement of plant growth parameters results not only from the direct effects these bioinoculants impose on them but also from their non-target effects. The ability of bioinoculants to reduce the application of chemicals for obtaining optimum yield of legume appears to be of great ecological and economic importance. In the present study, we compared the influence of seed inoculation of *Cajanus cajan* with a microbial consortium, comprising *Bacillus megaterium*, *Pseudomonas fluorescens* and *Trichoderma harzianum*, with that of application of chemical fertilizers on plant’s growth parameters and its rhizospheric microbial communities. Real-time PCR assay was carried out to target the structure (16S rRNA) and function (nitrogen cycle) of rhizospheric microbiota, using both DNA and RNA as markers. The results showed that the microbial consortium was the most efficient in increasing grain yield (2.5-fold), even better than the recommended dose of chemical fertilizers (by 1.2-fold) and showed enhancement in *nif*H and *amo*A transcripts by 2.7- and 2.0-fold, respectively. No adverse effects of bioinoculants' application were observed over the rhizospheric microbial community, rendering the consortium to be safe for release in agricultural fields.

## Introduction

In the present century the increasing population has pressurized agriculture in two manners, first the ardent need to meet the demand for food grains, and second to meet these demands in an environmental friendly fashion. A promising answer to this challenge is the implementation of sustainable agriculture that involves the utilization of an array of techniques as organic farming, application of bioinoculants, and modified cropping systems. Although the use of chemical fertilizers has led to an enhancement in improved agricultural production, several major health and environment related concerns have been associated with them [1]. Also, the widespread problem of plant pathogens makes it necessary to explore new methods to secure plant growth and their health [2]. Therefore, there has been an increased popularity of bioinoculants owing to their soil or plant origin.

Various studies have been reported highlighting the beneficial effects of bioinoculants in relation to enhanced crop productivity and better plant health [3–5]. However, introduction of microbial inoculants into soil in higher numbers must also induce, at least a transient, disturbance in the equilibrium of soil microbial communities. Such effects of the introduced bioinoculants on soil microbial composition, other than their target organisms, or on biogeochemical cycles are known as non-target effects [6]. In agriculture, very little effort has actually gone into analyzing the non-target effects of the introduced bioinoculants, apart from their direct effects, resulting into better plant growth. In addition to rhizosphere, the microbial inoculants colonize the rhizoplane and the roots as well [7–9]. Herschkowitz *et al*. [10] studied the effect of *Azospirillum brasilense* strains on *Zea mays* roots and on bacterial community in rhizoplane–endorhizosphere. They reported a decrease in bacterial complexity as compared to rhizosphere. Another group studied the effect of a commercial product containing spores of *Bacillus amyloliquefaciens* FZB42 on lettuce plants under infected condition by *Rhizoctonia solani*, together with its impact on the indigenous rhizosphere bacterial community in field and pot experiments [11]. They found effective control of bottom rot disease with the application of the inoculant on the field grown lettuce without exerting any noticeable effect on rhizospheric bacterial community.

The present study aims to assess and compare the target as well as non-target effects of a microbial consortium, comprising three selected bioinoculants, (*Bacillus megaterium*, *Pseudomonas fluorescens* and *Trichoderma harzianum*), which had been earlier reported to result in significant enhancement on various parameters of *Cajanus cajan* [4] with that of chemical fertilizers at recommended dose, using cultivation-independent approach. The hypothesis underlying the project was that the positive effects of bioinoculants on plant's growth and grain yield is a summation of target and non-target (on other members of the rhizospheric resident microbiota) effects. This may result due to their synergistic or inhibitory interactions with the resident microbial populations leading to enhanced or suppressed microbial processes like N cycle, hence causing changes in soil nutrient status and finally plant growth.

*Cajanus cajan*, commonly known as pigeon pea, is known to be the major grain crop of semi-arid tropics. It has high protein content, and is therefore commonly used as a substitute for meat in a largely vegetarian population in India. The variety UPAS-120 is an extra early maturing variety. The bioinoculants employed in the present study were strategically chosen on the basis of their performance in terms of enhancement of plant growth and grain yield in various crops. *B*. *megaterium* MTCC 453 (ATCC 14945) is known to show biocontrol property against rhizospheric nematodes through the production of a major extracellular neutral protease [12, 13]. *P*. *fluorescens* MTCC 9768, isolated from rhizospheric soil of pea plant, acts as both biocontrol agent and plant growth promoting rhizobacterium with the properties including phosphate solubilization, production of siderophores, HCN, and IAA as suggested by Mishra *et al*. [14]. *Trichoderma harzianum* MTCC 801, isolated from sugar beet field, is commonly used as antagonist in biocontrol of some important plant pathogenic soil-borne fungi [15].

Since nitrogen is one of the most crucial elements required for plant growth promotion, it is necessary to understand the non-target effects of any agricultural amendment (bioinoculants or chemical fertilizers) on key rhizospheric microbial communities involved in nitrogen turnover in the soil [16, 17]. Therefore, various steps of nitrogen cycle, including nitrogen fixation, nitrification and denitrification, were targeted as a response to introduction of bioinoculants and chemical fertilizers. Resident as well as active microbial populations were analyzed by the co-extraction of DNA and RNA. Abundance of resident and active total bacteria, and the communities involved in nitrogen cycle were analyzed using real-time PCR assay for quantitative analysis.

## Materials and Methods

### Physico-chemical properties of the soil

Clayey loam soil with clay (35–40%), loam (25–30%) and sand (20–25%), 0.42% organic matter content, 7.4 pH (in water), and 0.06 dS.m^−1^ electrical conductivity was used in the study. It had nitrogen, phosphorus, potassium and iron content of 136.5, 98.5, 176.25, and 14.2 mg kg^−1^, respectively.

### Microbial strains and compatibility assay

Two bacterial and one fungal strain were used as bioinoculants for the study, viz. *Bacillus megaterium* MTCC 453, *Pseudomonas fluorescens* MTCC 9768 and *Trichoderma harzianum* MTCC 801 (procured from the Institute of Microbial Technology, Chandigarh, India). Glycerol stocks of *B*. *megaterium* and *P*. *fluorescens* cultures were maintained at −20°C in Sperber medium [18] and King’s B medium [19], respectively. *T*. *harzianum* was maintained in Rose Bengal Chloramphenicol medium [20]. The three microbial strains were checked for their compatibility with each other before being used as bioinoculants, using cross streak assay method [21].

### Preparation of formulation

The culture broths containing 1 × 10^8^ cfu or spores ml^−1^ of each microbial culture were used for the preparation of formulations. While the cfu was counted for *Pseudomonas* and *Bacillus*, spores were counted for the fungal member, *Trichoderma*. Talcum powder was sterilized three-times in autoclave bags at 121°C at about 15 psi pressures. To prepare hundred gram of inorganic carrier based formulation, 80 g of sterilized talcum powder (Starke & Co. Pvt Ltd, New Delhi, India), 18 ml of microbial suspension (6 ml of each microbial culture), 1 ml of glycerol (50% w/v) and 1 ml of carboxymethylcellulose (CMC) solution (0.1 mg/ml) as adhesive, were mixed under sterile conditions [22]. The product was then shade dried to reduce the moisture content. The formulation contained 2 × 10^7^ cfu g^−1^ of each bioinoculant.

### Seed sterilization, bacterization and sowing

Seeds of an early maturing variety of *Cajanus cajan* (pigeon pea), UPAS 120, were procured from National Seed Corporation, IARI, New Delhi, India. The seeds were surface-sterilized [4] and then soaked in autoclaved water and kept overnight. Seeds of approximately similar shape and size were chosen for seed bacterization (by visual observation and by passing through 0.8 cm mesh size coarse sieve). Seed bacterization was done by mixing a fixed number of seeds with the formulations prepared.

Cfu per seed were found to be ~1 × 10^6^ seed^−1^ for each of the three bioinoculants (using serial dilution and plate count method). Treatments with triple inoculation and chemical fertilizers (at recommended dose of N @ 8 kg/acre, and P @ 16 kg/acre (http://agriharyana.nic.in/variouscrops.htm)) were set in 16 pots each (4 sampling points × 4 replicates) of approximately 40 cm diameter filled with soil mentioned above. Host specific *Rhizobium* strain was added to all the treatments i.e. C, BPT and NP; except US (unplanted/bulk soil without any seed or plant), and mixed well as recommended by the manufacturer (procured from IARI, New Delhi). Besides, pots with uninoculated seeds and bulk / unplanted soil were also maintained accordingly (4 sampling points × 4 replicates) under same experimental conditions. The nomenclature used for the treatments was: BPT as microbial consortium (*B*. *megaterium* MTCC 453 + *P*. *fluorescens* MTCC 9768 + *T*. *harzianum* MTCC 801); C as control i.e. seeds without any inoculation; US as unplanted (and un-inoculated) soil, and NP, i.e. chemical fertilizers. Seeds were sown in pots at a depth of about 4–5 cm. Completely randomized block design was used for the experiment. The pots were kept under sunlight (approximately 16/8 photoperiod) and temperature ranging from 23°C to 34°C. The pots were irrigated at regular intervals to maintain constant moisture level in the soil (approximately 14%).

### Sampling

Vegetative stage (1 month after seed sowing), pre-flowering (2 months after seed sowing), flowering (3 months after seed sowing) and maturity (4 months after seed sowing) stages were selected as sampling points. At each time point four randomly selected whole *C*. *cajan* plants were sampled for each treatment and control. The roots of the uprooted plants were shaken vigorously to collect the soil adhering to roots, without damaging the root and root nodules, and this soil was termed as “rhizosphere soil”. Rhizosphere samples was stored at −20°C (after shock freezing in liquid nitrogen) for cultivation-independent studies.

### Plant growth experiment

The growth parameters viz. shoot length, root length, composite dry weights of stems and roots, and grain yield per plant, were measured at each time point. Length of shoot was measured from the base of stem to its tip, while root length was measured from its point of attachment on stem base to the tip of the tap root. The composite dry weights of stems and roots were measured after drying in oven at 70°C for 24 h. Grains were collected from the pods and the grain mass per plant was recorded.

### Total nucleic acid extraction and cDNA synthesis

Total nucleic acid extraction from rhizosphere soil samples, stored at -20°C, was performed [23]. Half of the extract was treated with DNase I, RNase-free enzyme (Thermo Scientific, USA), prior to cDNA synthesis, to remove the genomic DNA co-extracted with RNA, according to the manufacturer’s protocol. For prevention of the action of RNases all the glassware were rinsed with diethylene pyrocarbonate (DEPC) treated water and then incubated overnight at 200°C. Reverse transcription of RNA was performed, with random hexamer, in a final reaction volume of 20 μl using RevertAid First strand cDNA synthesis Kit (Thermo Scientific, USA). The reaction mixture was incubated for 5 min at 25°C, followed by 60 min at 42°C. The reaction was terminated by heating at 70°C for 5 min. The product was then stored at -20°C for further applications.

### Real-time PCR assay

Quantitative PCR (qPCR) assay was performed for both DNA and cDNA to quantify the resident as well as active microbiota, respectively in the plant rhizosphere. The abundance of total bacteria in the rhizosphere samples was assessed using 16S rRNA primer-based qPCR assay employing specific primers (341F and 534R) and PCR conditions as mentioned by López-Gutiérrez *et al*. [24].

Genes targeting nitrogen fixation (*nif*H), ammonia oxidation (*amo*A) and denitrification (*nar*G, *nir*K and *nir*S) were used as molecular markers to monitor the abundances of nitrogen fixers, ammonia oxidizers and denitrifiers, respectively, using the primers and PCR conditions mentioned earlier [4]. qPCR assays were carried out in polypropylene 96-well plates with the CFX96 Touch Real-Time PCR Detection System (Bio-Rad, Hercules, CA, USA) by using SYBR green as the detection system in a reaction mixture of 15 μl containing 0.5 μM of each primer; 7.5 μl of 2X SsoFast EvaGreen Supermix (Bio-Rad, Hercules, CA, USA), and 0.5 μl of diluted template corresponding to 10 ng of total DNA. A minimum of two independent qPCR assays were performed on each of the four biological replicate samples and an average technical variability of 16% was observed. Standard curves were obtained with serial dilutions of a known amount of the plasmid DNA, containing a fragment of the targeted gene (*nir*K: *Sinorhizobium meliloti*; *nir*S: *Pseudomonas aeruginosa*; *nif*H: *Frankia alni*; *nar*G: *Pseudomonas aeruginosa*) (r^2^ > 0.885 for all assays and intercepts ranged between 25.6 and 42.4 depending on the targeted genes). PCR efficiency for the different assays ranged between 84 and 106%. Possible effects of qPCR inhibitors, co-extracted during nucleic acid extraction, was tested by mixing a known amount of pGEM-T plasmid with the soil DNA extracts or water with the plasmid-specific T7 and SP6 primers, before running qPCR assays. No Template Controls (NTC) gave null or negligible values.

### Statistical analysis

The experiments were carried out in a completely randomized block design. Standard deviations for each treatment were calculated. The data were subjected to analysis of variance (ANOVA) using SPSS Statistical System (SPSS 16.0 for Windows) with dependent variables as the respective ‘plant growth parameter’ or ‘gene copy number’ and independent variable as either ‘treatment’ or ‘time point’. Comparison between means was made using Duncan’s multiple range test (DMRT) at p < 0.05 [25]. To visualize the rhizospheric community shift for structural and functional genes under various treatments, non-metric multidimensional scaling (NMS) was performed with r^2^ >0.9 and the stress value <0.01 [26]. This analysis was performed with ALSCAL program of SPSS software version 16.0.

## Results

For all the four treatments, sampling of rhizosphere soil and *C*. *cajan* plants was performed at four different growth stages of the plant. After one month of sowing there was a decrease in soil pH from 7.35 to 6.8 only in un-planted soil. Soil pH remained stable (approximately 7.1–7.3) in all other treatments.

### Effect of treatments on plant growth parameters

**Fig 1:**
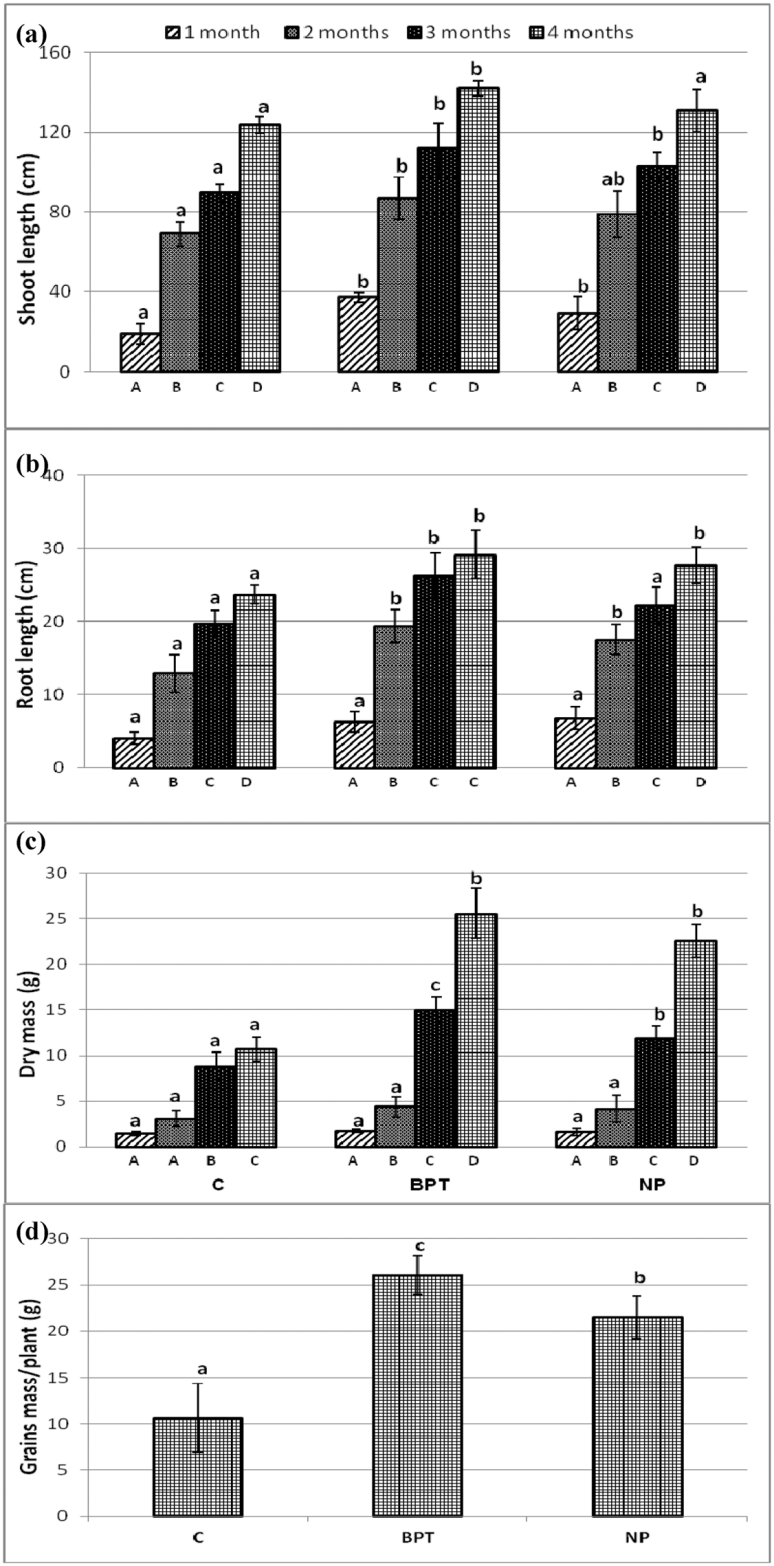
Growth parameters of *Cajanus cajan* var. UPAS 120 (a) shoot length, (b) root length, (c) dry mass, (d) grain yield, at four time points, viz. vegetative growth stage (1 month after sowing), pre-flowering stage (2 months after sowing), flowering stage (3 months after sowing), and maturity stage (4 months after sowing). Significantly different values (p < 0.05) between different sampling time points in the same treatment are marked by uppercase letters (A to D) under the columns, and among treatments for the same time point are marked by lower case letters (a to c). Error bars represent standard deviations (*n* = 3).

The effect of time point and treatment was assessed on growth parameters of *C*. *cajan* at the four developmental stages of the plant. As expected, all plant parameters showed enhancement with the growth of plant, being maximum at the maturity stage of the plant. Also, it was found that the treatments with the microbial consortium and/or chemical fertilizers showed enhancement of plant parameters (with the exception of chemical fertilizer exerting no effect on shoot length) over control at the maturity stage of the plant (Fig 1). However, the maximum enhancement in all the plant parameters, over control plants, was observed with the application of microbial consortium, which was better than the recommended dose of the chemical fertilizers. The application of the microbial consortium showed an increase in shoot length (1.2-fold), root length (1.3-fold), dry mass (2.4-fold) and grain yield (2.5-fold) of *C*. *cajan* plants during harvest. The use of chemical fertilizers (NP) also led to improvement in plant parameters over control but upto a lesser extent than that with the microbial consortium, viz. root length (1.0-fold), dry mass (2.0-fold), and grain yield (2.0-fold).

Shifts in plant growth parameters based on biometric observations were visualized by non-metric multidimensional scaling (NMS) analysis under different treatments and absolute quantity matrix was generated based on the growth parameters data (S1 Fig). Plant growth parameters grouped separately in response to different treatments. A remarkable shift in all plant parameters analyzed with the application of bioinoculants (BPT) and chemical fertilizers (NP) was demonstrated when compared to control (C). Another shift was observed with BPT treatment over NP, which was reflected by significant increase in grain yield (1.2-fold) with BPT treatment as compared to NP (Fig 1a–1d).

### Effect of treatments on resident and active bacterial population in *C*. *cajan* rhizosphere

**Fig 2:**
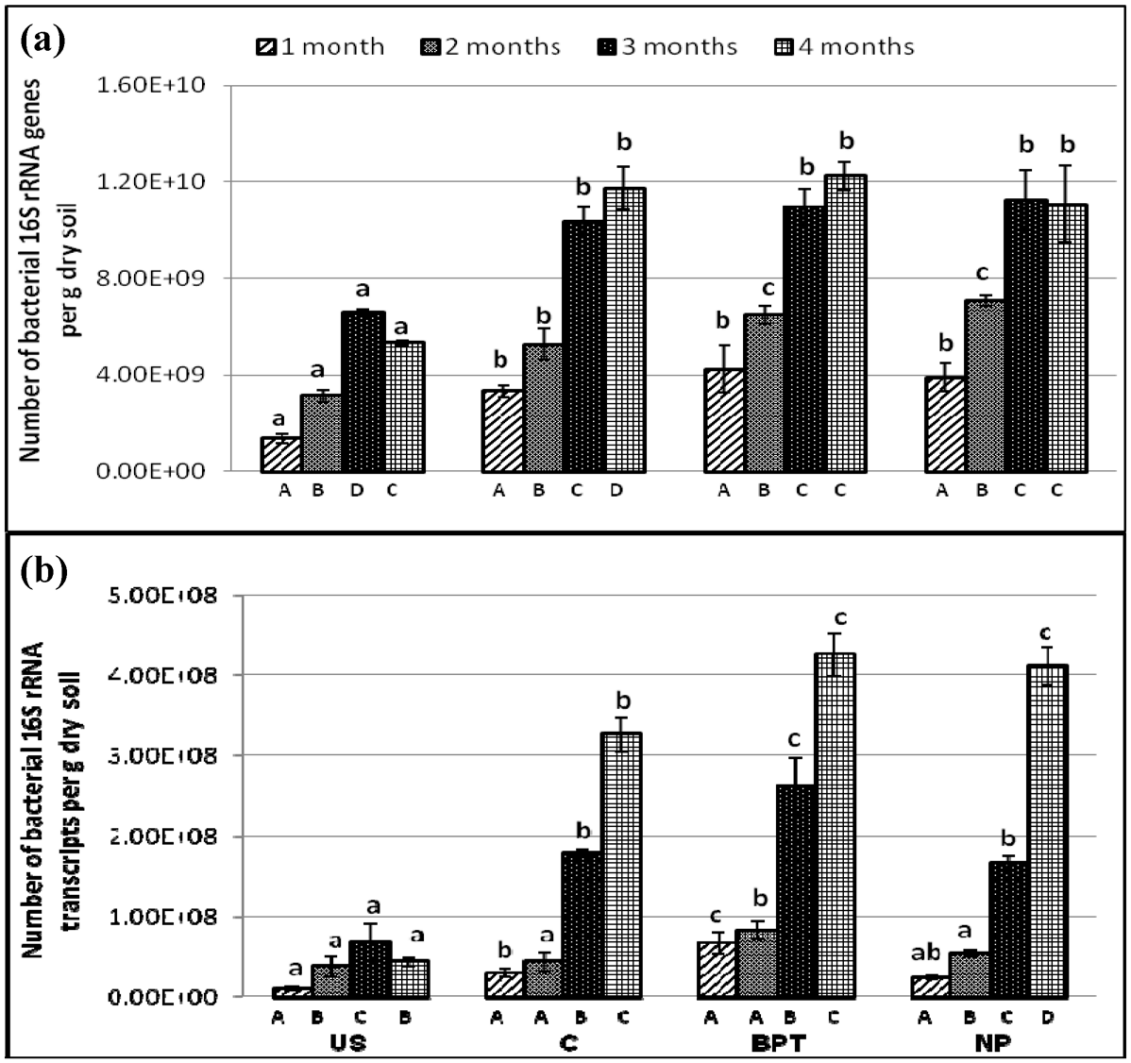
Abundance (copies per g dry soil) of bacterial 16S rRNA (a) genes (b) transcripts under different treatments at four time points. Error bars represent standard deviations (*n* = 3). Significantly different values (p < 0.05) between different sampling time points in the same treatment are marked by capital letters (A to D) under the columns, and between different treatments for the same time point are marked by lower case letters (a to c).

The variations in abundance of resident and active bacterial members in the bulk as well as *C*. *cajan* rhizosphere soil samples were monitored using qPCR assay. Products of 177 bp from 16S rRNA genes and transcripts were successfully amplified from the bulk soil (US) as well as rhizosphere of treated (BPT, NP) and control (C) *C*. *cajan* plants, ranging from 1.3 × 10^9^ to 1.2 × 10^10^ copies of genes, and 1.1 × 10^7^ to 4.2 × 10^8^ copies of transcripts per gram dry soil (Fig 2).

Rhizosphere effect was evidenced at both gene and transcript levels as control (C) showed increased abundance over bulk soil (US). It was observed that the population size of the bacterial communities at both DNA and RNA levels was stimulated in the rhizosphere of *C*. *cajan* in all treatments at 3rd and 4th time points (Fig 2). However, no significant effect of treatment (BPT or NP) was observed with respect to control (C) over the abundance of total resident bacterial population (in terms of 16S rRNA gene copies) showing it to be a stable structure (Fig 2a).

In case of active bacterial population (in terms of 16S rRNA transcript as marker), both plant age and the treatments (BPT and NP) showed significant enhancements over control plants, as evidenced by an increased number of transcripts at 3^rd^ and 4^th^ time points (Fig 2b). There was an increase in 16S rRNA transcripts with the application of microbial consortium (BPT) over control at all plant growth stages, the transcript copies being maximum at 4^th^ time point (by 1.3- fold). Similar level of increase in 16S rRNA transcripts’ number was observed with chemical fertilizers also (1.3- fold) but only at 4^th^ time point.

The absolute matrix showed a noticeable shift in the abundance of resident as well as active bacterial members in rhizosphere samples as compared to bulk soil (S2 Fig), which can be attributed to rhizosphere effect. A smaller shift was observed in resident bacterial members in the treated as well as control rhizosphere samples, showing no effect of treatments. This was also reflected by the stable structure observed in Fig 2a. However, a wide shift was seen in the active bacterial members as a response to different treatments (BPT and NP) when compared to control (C).

### Abundance of functional genes and transcripts involved in nitrogen cycling

**Fig 3:**
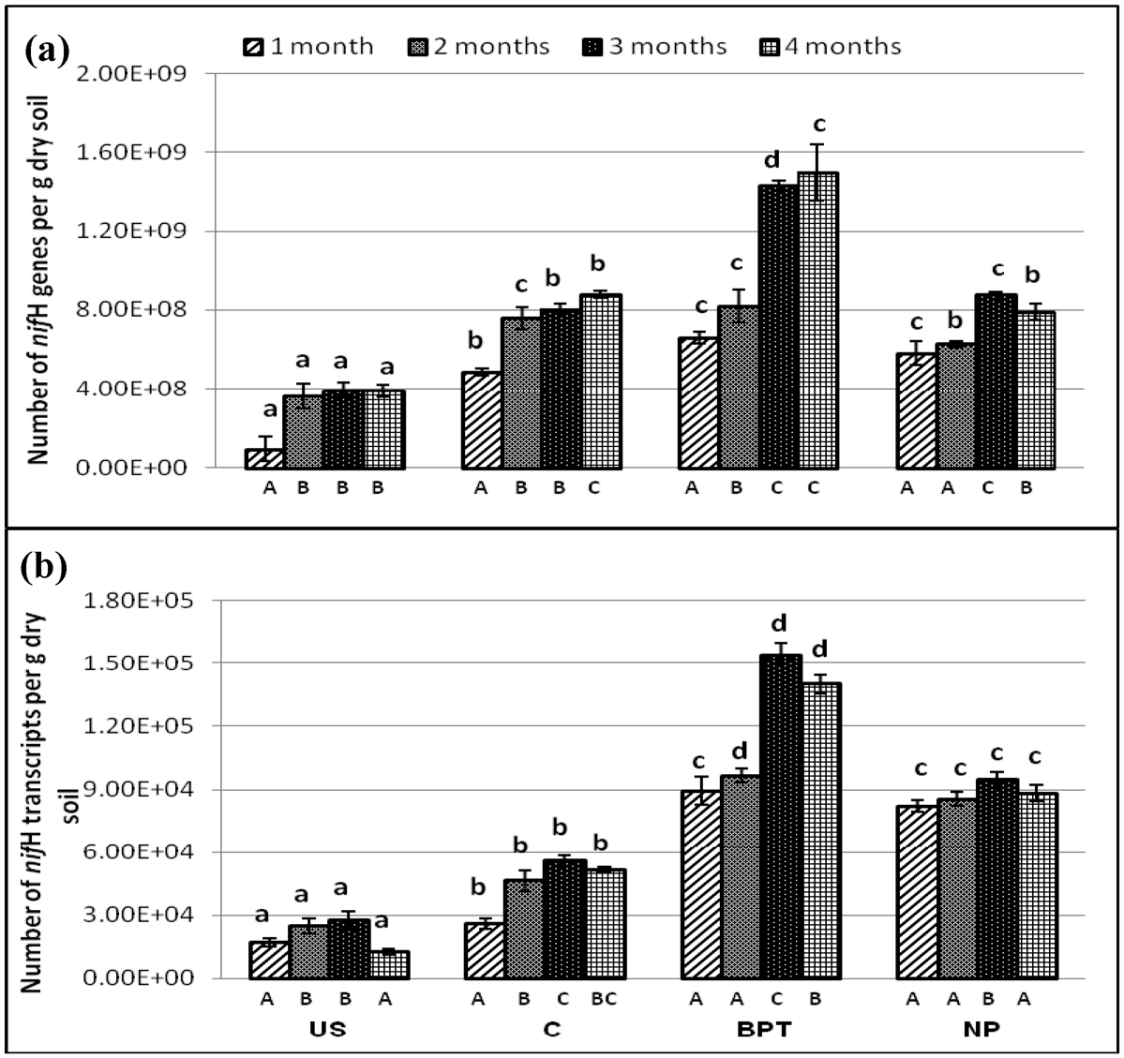
Abundance (copies per g dry soil) of (a) genes (b) transcripts of nitrogenase (*nif*H) under different treatments at four time points. Error bars represent standard deviations (*n* = 3). Significantly different values (p < 0.05) between different sampling time points in the same treatment are marked by capital letters (A to C) under the columns, and between different treatments for the same time point are marked by lower case letters (a to d).

**Fig 4:**
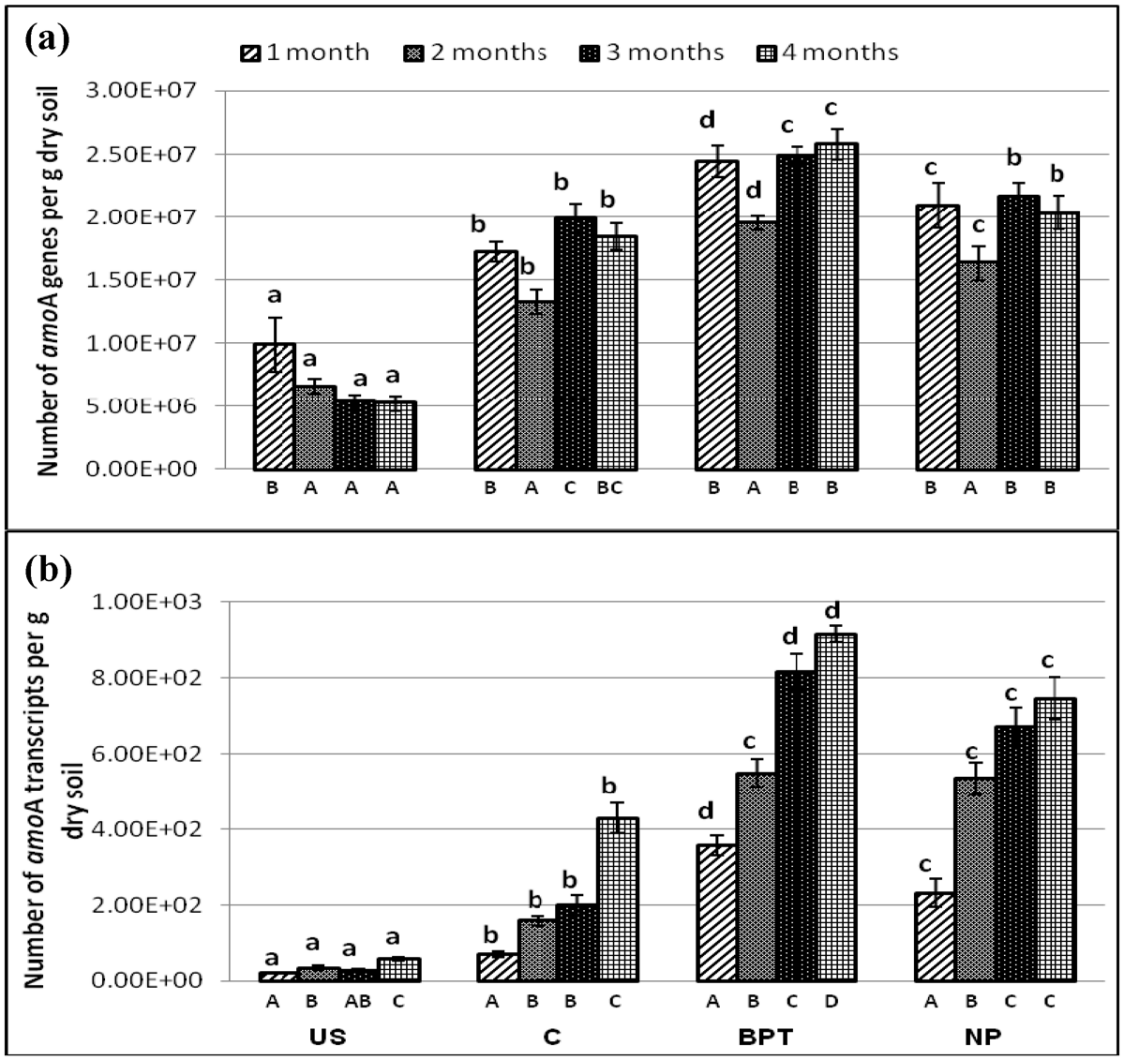
Abundance (copies per g dry soil) of (a) genes (b) transcripts of ammonia-monooxygenase (*amo*A) under different treatments at four time points. Error bars represent standard deviations (*n* = 3). Significantly different values (p < 0.05) between different sampling time points in the same treatment are marked by capital letters (A to D) under the columns, and between different treatments for the same time point are marked by lower case letters (a to d).

**Fig 5:**
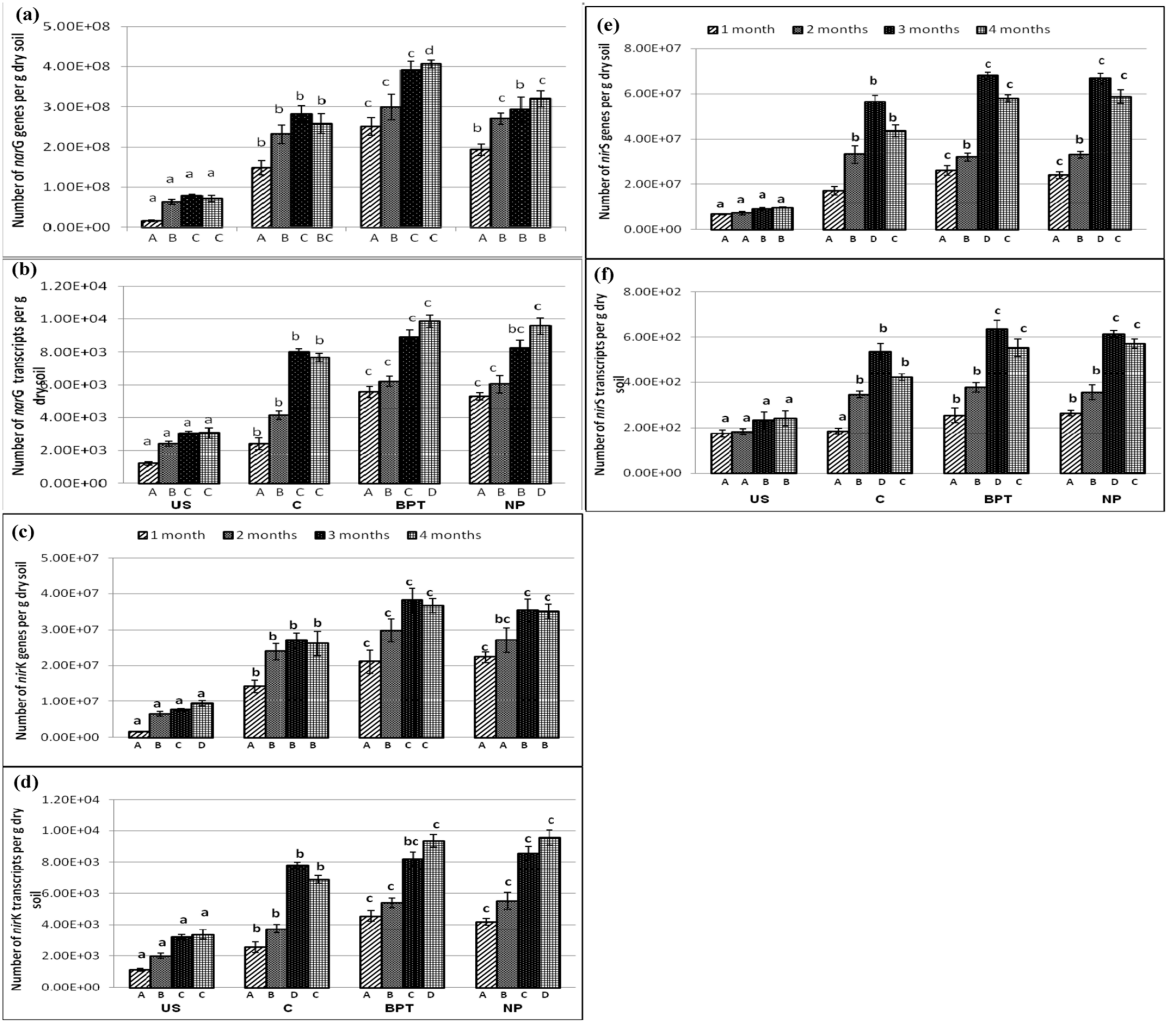
Abundance (copies per g dry soil) of (a) genes and (b) transcripts of membrane-bound nitrate reductase (*nar*G); (c) genes and (d) transcripts of Cu- containing nitrite reductase (*nir*K); (e) genes and (f) transcripts of cytochrome cd1- containing nitrite reductase (*nir*S) under different treatments at four time points. Error bars represent standard deviations (*n* = 3). Significantly different values (p < 0.05) between different sampling time points in the same treatment are marked by capital letters (A to D) under the columns, and between different treatments for the same time point are marked by lower case letters (a to c).

A series of qPCR and q-RT-PCR assays were performed to investigate the variations in the levels of *nif*H (nitrogen fixers), *amo*A (nitrifiers), *nar*G, *nir*K and *nir*S (denitrifiers) gene and transcript abundance, respectively, in the soil samples. The assayed microbial genes showed a significant response to different sampling time points, as well as treatments. Effect of plant roots (rhizosphere effect) was also observed at both gene and transcript levels in the N cycle markers at all plant growth stages (Figs 3, 4, 5).

### Effect on abundance of *nif*H genes and transcripts

The soil samples taken at different time points contained *nif*H genes and transcripts ranging from 9.4 × 10^7^ to 1.5 × 10^9^ genes and 1.3 × 10^4^ to 1.5 × 10^5^ transcripts per gram dry soil, respectively.

Maximum abundance of *nif*H gene as well as transcript was with BPT treatment towards the later stages of plant development, even higher than the recommended dose of chemical fertilizers by 1.9- and 1.6-fold, respectively (Fig 3). A fold increase of 1.7 and 2.7 in *nif*H genes and transcripts, respectively, was found with microbial consortium over control at maturity stage. The chemical fertilizers did not show any effect on *nif*H genes while *nif*H transcripts showed enhancement by 1.7-fold over control at 4^th^ time point.

The clustering in NMS showed that the BPT treated rhizosphere for both resident and active nitrogenase (*nif*H) containing bacterial members lied separately from other treatments (S3 Fig). The resident nitrogen fixing bacteria in both fertilizer treated as well as control rhizosphere grouped together, exhibiting no effect of chemical fertilizers on the abundance of nitrogen fixers. However, the activity of nitrogen fixing bacteria was influenced by the chemical fertilizer's application also as a remarkable shift was observed in NP treated and control rhizosphere samples in terms of *nif*H transcripts per g dry soil.

### Amplification of *amo*A genes and transcripts

The *amo*A genes and transcripts ranged from 5.2 × 10^6^ to 2.6 × 10^7^ and 2.1 × 10^1^ to 9.2 × 10^2^ copies per gram dry soil, respectively. *amo*A genes were in high numbers at all plant growth stages except 2^nd^ stage, i.e. (pre-flowering stage) while *amo*A transcripts were highest at the final stage only (Fig 4). Maximum stimulation in *amo*A genes and transcripts occurred with BPT treatment over C by 1.4- and 2-fold, respectively at final stage of plant growth. Similar to *nif*H amplification, no effect of chemical fertilizers was seen over *amo*A genes while *amo*A transcripts were enhanced by 1.7-fold over C at 4^th^ time point.

Subjecting the data to NMS, rhizosphere effect was conspicuous with a wide shift in *amo*A gene and transcripts numbers in the rhizosphere samples (C) and the bulk soil samples (US), which can be attributed to the higher diversity of microbes thriving on root exudates (S4 Fig). A small shift was observed between *amo*A gene copies in fertilizer treatment and control rhizosphere samples, while *amo*A transcripts showed a noticeable shift amongst the two, highlighting the effect of chemical fertilizers on active nitrifiers only. BPT formed a separate group for both *amo*A genes and transcripts, showing maximum response on nitrifying population to the application of bioinoculants.

### Effect on abundance of *nar*G, *nir*K and *nir*S genes and transcripts

The *nar*G genes and transcripts ranged from 1.64×10^7^ to 4.07×10^8^ and 1.21×10^3^ to 9.86×10^3^ copies per g dry soil (Fig 5a and 5b), respectively. Both *nar*G genes and transcripts were the most abundant at 3^rd^ and 4^th^ time points, respectively, with maximum number being at the maturity stage in BPT treated rhizospheric soil samples. The application of bioinoculants as microbial consortium led to an enhancement of 1.6-fold in *nar*G gene copies over control at 4^th^ time point, while it was only by 1.24-fold with chemical fertilizers. However, in case of *nar*G transcripts, both BPT and NP showed enhancement of approximately 1.3-fold over control soil samples at the 4^th^ time point.

The *nir*K genes and transcripts quantified in the collected soil samples ranged from 1.5 × 10^6^ to 3.8 × 10^7^ and 1.1 × 10^3^ to 9.5 × 10^3^ copies per gram dry soil, while *nir*S ranged from 6.7 × 10^6^ to 6.8 × 10^7^ genes and 1.7 × 10^2^ to 6.4 × 10^2^ transcripts per gram dry soil, respectively (Fig 5c–5f). The abundance and activity of *nir*K genes was highest at the flowering and maturity stages of plant development while *nir*S genes and transcripts were most abundant at the flowering stage only, in all the treatments including control. Both the denitrification genes showed significant response to the application of bioinoculants as well as chemical fertilizers, with an enhancement of 1.4- and 1.3-fold in *nir*K and *nir*S copies, respectively, over control at the last stage of plant growth.

Multidimensional clustering for the three denitrifying genes and transcripts, presented three separate groups, one containing both BPT and NP treated rhizosphere samples together, second containing C and third one having bulk soil (US), showing significant responses of *nar*G, *nir*K and *nir*S genes and transcripts to both the treatments (BPT and NP) as compared to control (S5 Fig).

## Discussion

Bioinoculants play an important role in sustainable agriculture. Though bioinoculants are currently gaining much popularity as alternative to synthetic fertilizers due to their display of minimal impacts on environment and high safety level, their mode of interaction with native plants and resident microbial community along with their mechanisms involved in various nutrient/biogeochemical cycles in fields is still a debatable question. Another major point of concern today is their survivability in the rhizosphere. However, as tracking of strains (unlike tracking of genera) is cumbersome, there are not many success stories on this aspect. The effects of the bioinoculants have thus been reported in the manuscript as a one-time application as seed-coating that is normally followed by farmers.

The present study assessed the non-target effects of three selected bioinoculants *B*. *megaterium* MTCC 453, *P*. *fluorescens* MTCC 9768 and *T*. *harzianum* MTCC 801, in the form of a microbial consortium, on the resident as well as active microbial community in *C*. *cajan* rhizosphere using cultivation–independent methods. In our previous studies [4, 5], we obtained best results in terms of plant parameters and grain yield with the consortium of *Bacillus megaterium*, *Pseudomonas fluorescens* and *Trichoderma harzianum* (BPT) when compared to mono (*B*. *megaterium* (B) or *P*. *fluorescens* (P) or *T*. *harzianum* (T) alone) and dual (B+P, B+T, P+T) inoculations. However, in the earlier reports no attempts were made to compare the effect of bioinoculants with that of the chemical fertilizers, with the study being restricted to the potential of the system (DNA was used as marker), while in the present study, RNA has also been used as molecular marker along with DNA for better resolution.

The bioinoculants employed in the present study promoted growth of *C*. *cajan* and beneficial rhizospheric microbial community by various mechanisms. *B*. *megaterium* is a well known phosphate solubilizer. Rajendran *et al*. [27] have reported enhanced nodulation and growth of pigeon pea plants by co-inoculation of *Bacillus* strains with *Rhizobium* spp. *P*. *fluorescens* is known for its biocontrol property and secretion of various plant-growth promoting substances including indole-3-acetic acid, siderophores and 2, 4-diacetyl phloroglucinol. Kumar *et al*. [28] reported improved quality and yield of *C*. *cajan* with reduced wilting caused by *Fusarium* pathogen by co-inoculation of *Sinorhizobium fredii* KCC5 and *Pseudomonas fluorescens* LPK2 with half dose of chemical fertilizers. *T*. *harzianum* provided protection to plants from pathogens with the release of lytic enzymes like chitinase and 1, 3-β glucanase against the cell wall of various plant pathogens. Niranjana *et al*. [29] have suggested increased seedling emergence and reduced *Fusarium* wilt disease incidence in pigeon pea plants with the use of fresh cultures of both *T*. *harzianum* and *P*. *fluorescens*. Besides these well known direct effects, certain unseen effects of these bioinoculants may also play a significant role in increasing soil fertility and thereby promoting plant growth such as nutrient mobilization, dead matter decomposition, induction of plant defense responses by the production of antibiotics, antifungal metabolites and phytostimulation [30].

Better results obtained with the application of bioinoculants as mixed microbial consortium over individual or dual inoculations have been reported in various other studies as well. Improved plant growth and enhanced biological nitrogen fixation with the mixed application of *Azospirillum lipoferum*, *B*. *megaterium* and *P*. *fluorescens* had been observed in *Oryza sativa* [31]. Such increased plant performance with enhanced efficacy and healthy effects on crops as a response to mixed inoculation may account for the observed synergistic effects among bioinoculants [31, 32]. The improved growth and grain yield of *C*. *cajan* with microbial consortium may also be related to higher nutrient uptake by plant roots due to the induced morphological changes like increased root number, hair, length and thickness with inoculation of phosphate solubilizing *Bacillus* sp. as reported by Raja *et al*. [31].

The enhancements in plant growth parameters obtained with the application of microbial consortium were even higher than those with the recommended doses of chemical fertilizers. The possible reasons behind this can be (i) the balancing of the nutrients in soil as a result of the different functions served by each component of the consortium, whereas only two specific nutrients (N and P) were added to the soil in case of chemical fertilizers (ii) the enhancement in plant growth and grain yield resulted not only from the direct effects of inoculated strain or added chemical fertilizer but also from the non-target effects of these inoculations leading to either induction or repression of soil native microbial populations.

When the number of bacterial 16S rRNA genes and transcripts were quantified, the effect of plant age was quite evident, whereas the effect of treatments was not observed at the gene level. This is in consistence with the observations of other workers who reported no significant changes in the structure of microbial community in plants’ rhizosphere as a response to the application of bioinoculants [33, 34]. One possible explanation for this stable-looking bacterial community structure can be the redundancy between different bacterial strains in the soil, i.e., loss of one bacterial strain might have been compensated with an enhancement in other strain, thereby maintaining the total population abundance constant [35]. Contrary to the results observed at the gene level, the impact of treatments was evident at the transcript level, which may be attributed to the fact that RNA is a more sensitive marker and even small fluctuations, which are not evident at DNA level, are highlighted at the transcripts level. It has to be, however, noted that genes encoding the 5S, 16S, and 23S rRNAs are typically organized into an operon in members of the domain Bacteria with the number of rRNA operons in bacterial genomes being in the range from 1 to 15 [36, 37]. Hence, a direct correlation between the number of genes and abundance of bacteria has not been attempted in the study.

To understand the nitrogen turnover in *C*. *cajan* rhizosphere as a response to the application of bioinoculants and chemical fertilizers, qPCR assays were performed targeting the genes and the transcripts at various steps of nitrogen cycling. It was observed that both the plant growth stage as well as the agricultural amendments strongly influenced the microbial guild involved in N cycle. Also the range of *nif*H transcripts observed in the present study was higher than other N cycle marker transcripts. Nitrogen fixation is one of the most crucial steps of nitrogen cycle, leading to increase in fixed nitrogen into the soil. A possible justification for the maximum abundance of *nif*H genes and transcripts at the flowering stage of the plant could be the maximum rhizodeposition occurring at this stage thereby harboring most of the beneficial microbes (useful for flowering of the plant) in its rhizosphere [38]. This abundance was maintained till the plant attained maturity in BPT- treated rhizosphere samples, while it was reduced in control highlighting the enhanced nutritive status of the soil with the application of the bioinoculants. Our results are in accordance with Babić *et al*. [16] who found highest *nif*H gene copy numbers in alfalfa (*Medicago sativa* L.) rhizosphere at late flowering stage when treated with *Sinorhizobium meliloti* OS6. At the maturity stage, the chemical fertilizers did not show any effect on *nif*H genes, while *nif*H transcripts showed enhancement over C. Other researchers have also studied the impact of different levels of nitrogen fertilizer on *nif*H gene abundance and diversity in *Sorghum bicolor* rhizosphere using *nif*H PCR-DGGE and qPCR analyses [39, 40]. They concluded that the amount of applied nitrogen fertilizer had significant impact on the structure and abundance of diazotrophs, as application in high doses led to reduced *nif*H density. The strongest increase observed with the microbial consortium application can be related to the cumulative effects of the bioinoculants in the consortium.

Ammonia oxidation is an important step in nitrification for increasing nitrate availability in the soil. It was found from our study that both *amo*A genes and transcripts were strongly affected by the consortium, indicating towards the non-target effects of added bioinoculants as none of them carried *amo*A gene. As observed for *amo*A genes, the impact of chemical fertilizers was not prominent at DNA level, whereas activity of *amo*A genes was strongly influenced by NP treatment, which was also reflected by enhanced plant growth with NP treatment. It may be because the affected bacteria were too low in number but metabolically active. Other workers have also analyzed the effect of chemical fertilizers on *amo*A gene copies in soil samples. For example, He *et al*. [41] studied the impact of different combinations of fertilizers nitrogen (N), phosphorus (P) and potassium (K) on AOB and AOA abundance and composition, and found the highest AOB numbers in NPK + organic matter treatment followed by NPK and NP treatments.

Denitrification is a detrimental process for agricultural soil as it leads to loss of nitrogen from soil. Also, it plays an important role in the emission of greenhouse gas leading to global climatic changes. Similar to other genes, denitrifying genes and transcripts were strongly affected by the application of microbial consortium and chemical fertilizers, highlighting their non-target effects since none of the introduced strains carried the genes for denitrification. The range of denitrification genes found in this study was in accordance with earlier reports [42, 43]. The maximum abundance of denitrifying community at the flowering and maturity stages of the plant can be attributed to increased carbon availability at this stage due to maximum rhizodeposition during the flowering and fruiting stages of the plant [38]. Interestingly, the increased process of ammonia oxidation with BPT treatment led to increased production of nitrate, which is in line with the enhanced denitrification process with BPT treatment at the flowering and maturity stage of the plant. Similar observations were reported by Babić *et al*. [16] in alfalfa rhizosphere. On a first look, it is surprising that the abundance of genes is directly related to the substrate availability, which should be more correlated with actual microbial activities than to their potential. A possible reason for this could be the dominance of r-strategists in rhizosphere [44], which show quick response to the favorable environmental conditions and demonstrate high reproductive rates [16].

## Conclusion

Our study highlights the performance of a microbial consortium comprising three bioinoculants, namely *Bacillus megaterium*, *Pseudomonas fluorescens* and *Trichoderma harzianum* on *Cajanus cajan* and its rhizospheric microbial communities. The consortium was found to be more efficient in improving plant growth and grain yield, even better than the recommended dose of chemical fertilizers. A significant enhancement in transcripts for nitrogen fixation and nitrification processes indicated that the designed consortium had a positive effect on the crucial rhizospheric microbial processes, and therefore, can be termed as “safe” with respect to its impact on non-target rhizospheric microbial communities. Thus, the present study underscores the importance of understanding the non-target effects of bioinoculants, along with their direct effects, before their use in agriculture.

## Supporting Information

S1 Fig Shifts in plant growth parameters based on the biometric observations under different treatments by non-metric multidimensional scaling analysis. Squares = shoot length; stars = root length; triangles = dry mass; circle = grain yield. C = Control, BPT = *B*. *megaterium* + *P*. *fluorescens* + *T*. *harzianum*, and NP = chemical fertilizers.
(DOC)

S2 Fig Quantitative shifts in bacterial population abundance and activity measured by q-PCR under different treatments by non-metric multidimensional scaling analysis. Hollow circles = bacterial 16S rRNA genes per g dry soil; solid circles = bacterial 16S rRNA transcripts per g dry soil.
(DOC)

S3 Fig Quantitative shifts in *nif*H gene abundance and activity measured by q-PCR under different treatments by non-metric multidimensional scaling analysis. Hollow circles = *nif*H genes per g dry soil; solid circles = *nif*H transcripts per g dry soil.
(DOC)

S4 Fig Quantitative shifts in *amo*A gene abundance and activity measured by q-PCR under different treatments by non-metric multidimensional scaling analysis. Hollow circles = *amo*A genes per g dry soil; solid circles = *amo*A transcripts per g dry soil.
(DOC)

S5 Fig Quantitative shifts in (Figure A) *narG* (Figure B) *nir*K (Figure C) *nir*S gene abundance and activity measured by q-PCR under different treatments by non-metric multidimensional scaling analysis. Hollow circles = *nar*G/*nir*K/*nir*S genes per g dry soil; solid circles = *nar*G/*nir*K/*nir*S transcripts per g dry soil.
(DOC)
