# Supplementary figures and images for: Effect of Agricultural Amendments on Cajanus cajan (Pigeon Pea) and Its Rhizospheric Microbial Communities – A Comparison between Chemical Fertilizers and Bioinoculants
Source: PLoS One. 2015 Jul 31;10(7):e0132770. doi: 10.1371/journal.pone.0132770 (PMC4521884; doi:10.1371/journal.pone.0132770)

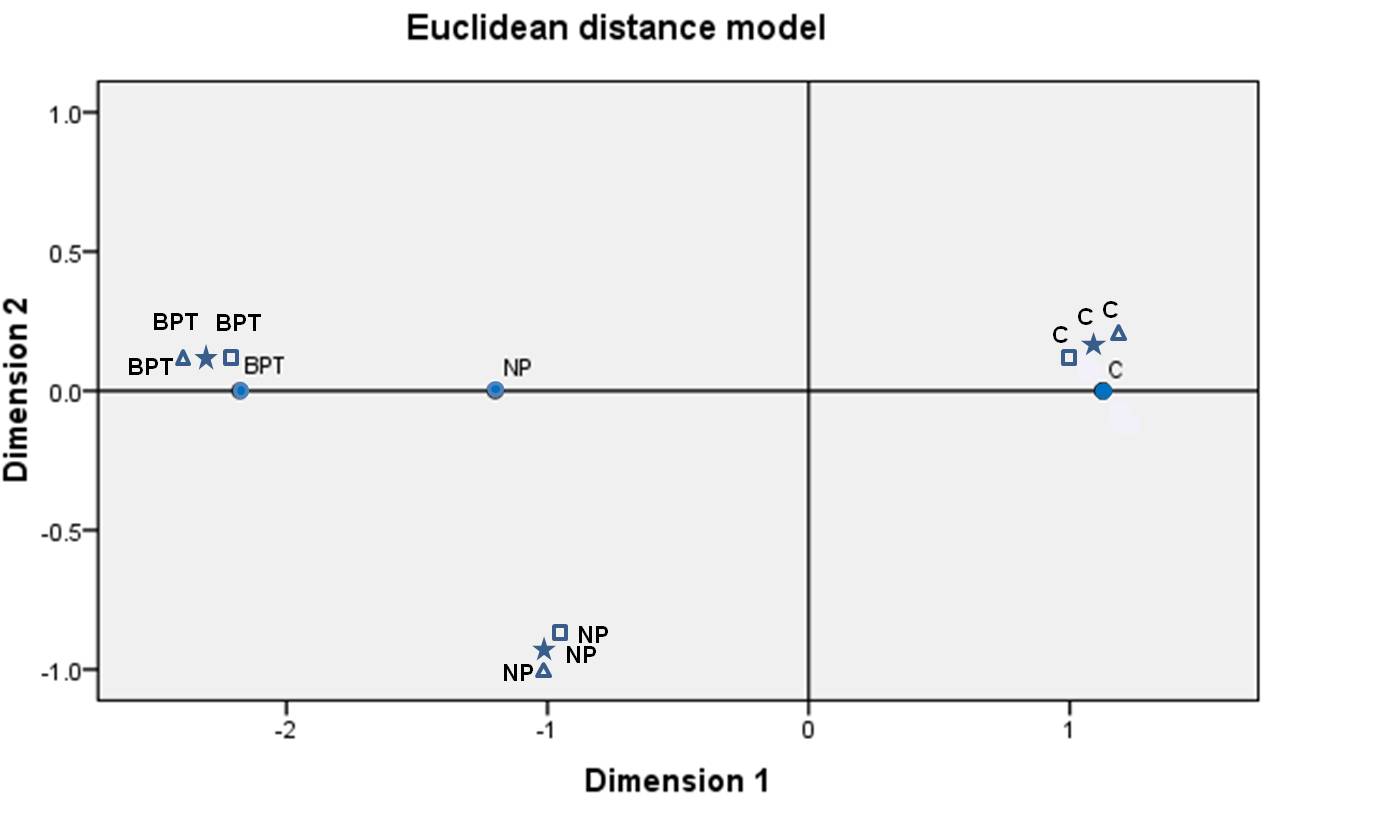


**S1 Fig**

Supplement: S1 Fig — Squares = shoot length; stars = root length; triangles = dry mass; circle = grain yield. C = Control, BPT = B. megaterium + P. fluorescens + T. harzianum, and NP = chemical fertilizers. (DOC) [file pone.0132770.s001.doc]

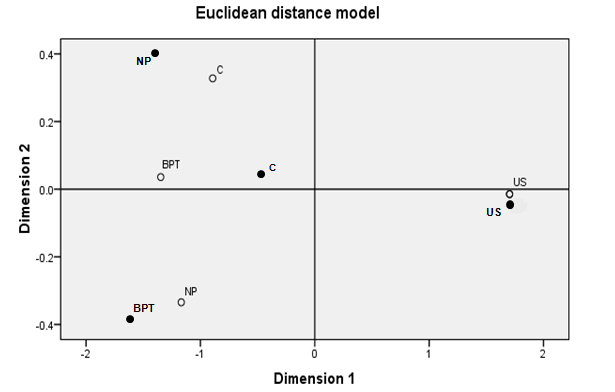


**S2 Fig**

**S5 Fig**

**(C)**

Supplement: S2 Fig — Hollow circles = bacterial 16S rRNA genes per g dry soil; solid circles = bacterial 16S rRNA transcripts per g dry soil. (DOC) [file pone.0132770.s002.doc]

**
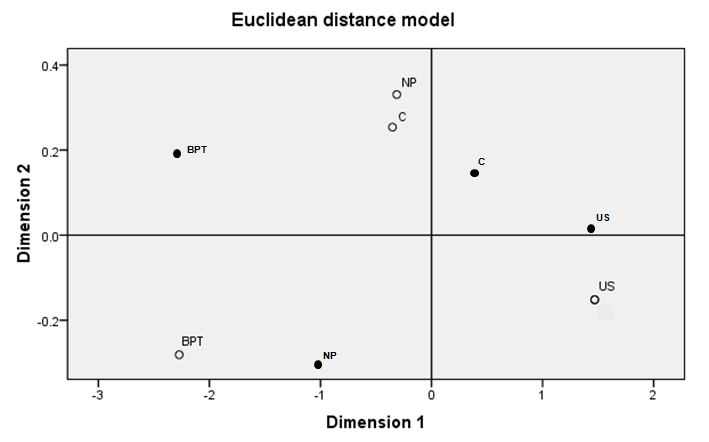
**

**S3 Fig**

**S5 Fig**

**(C)**

Supplement: S3 Fig — Hollow circles = nifH genes per g dry soil; solid circles = nifH transcripts per g dry soil. (DOC) [file pone.0132770.s003.doc]

**
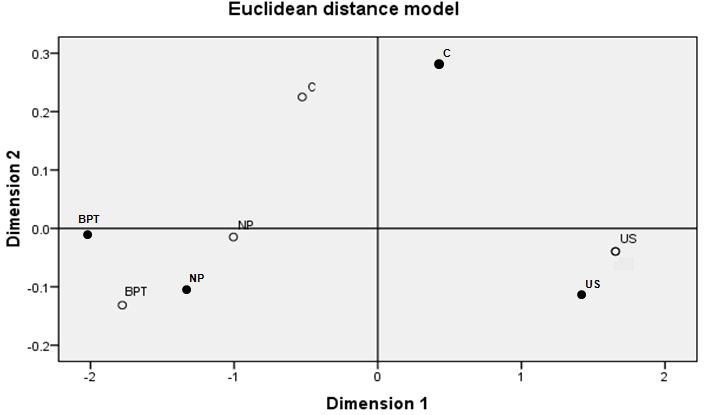
**

**S4 Fig**

**S5 Fig**

Supplement: S4 Fig — Hollow circles = amoA genes per g dry soil; solid circles = amoA transcripts per g dry soil. (DOC) [file pone.0132770.s004.doc]

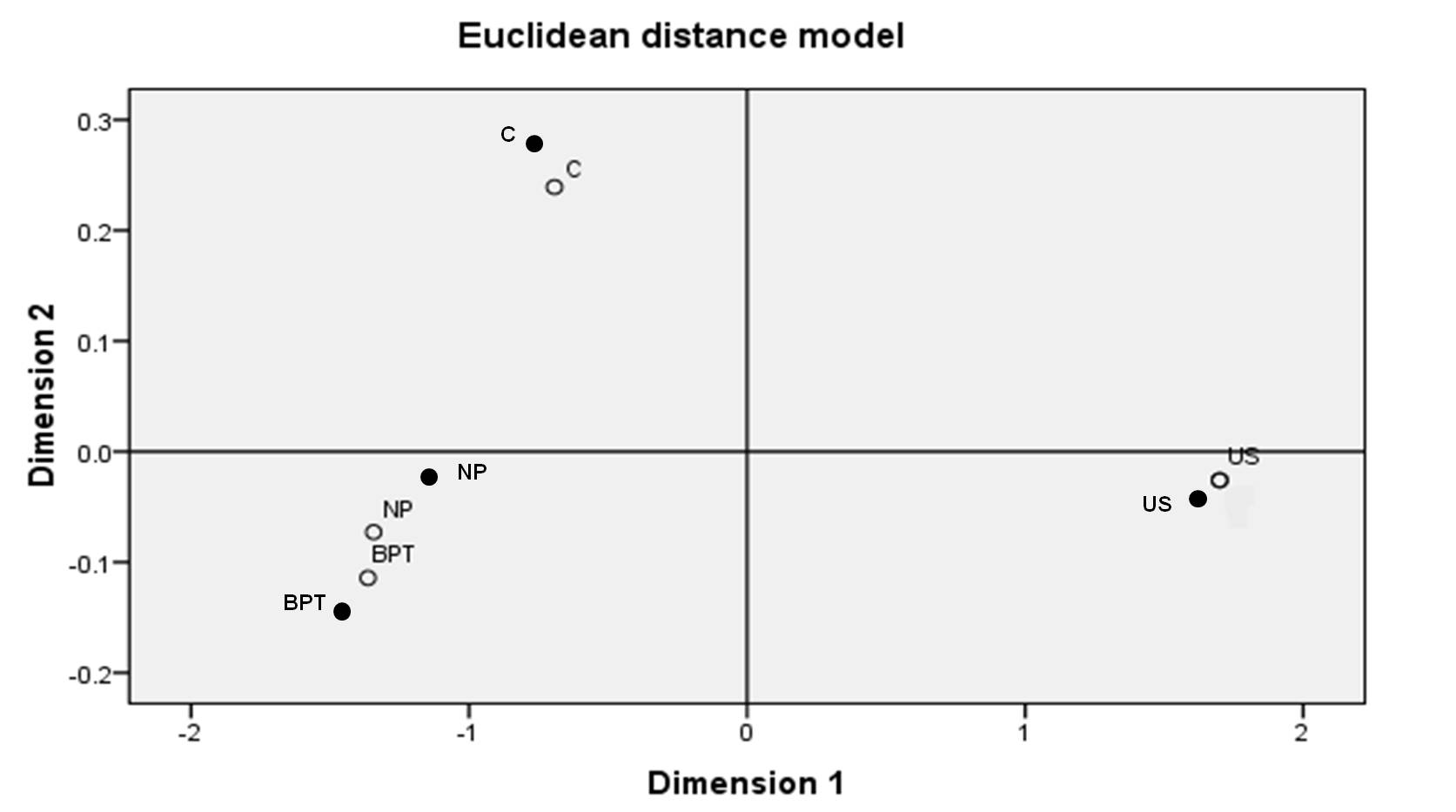

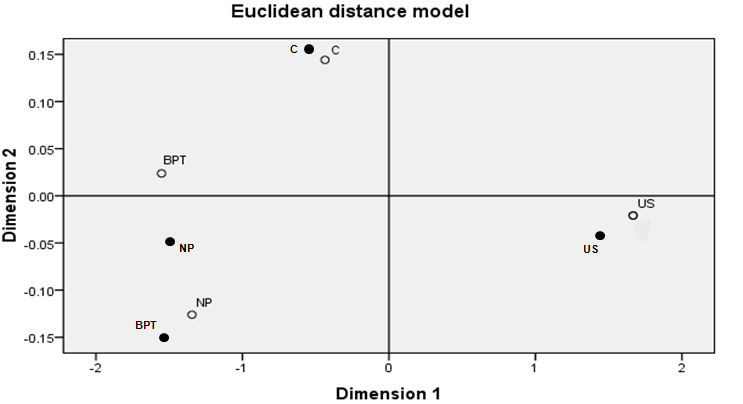

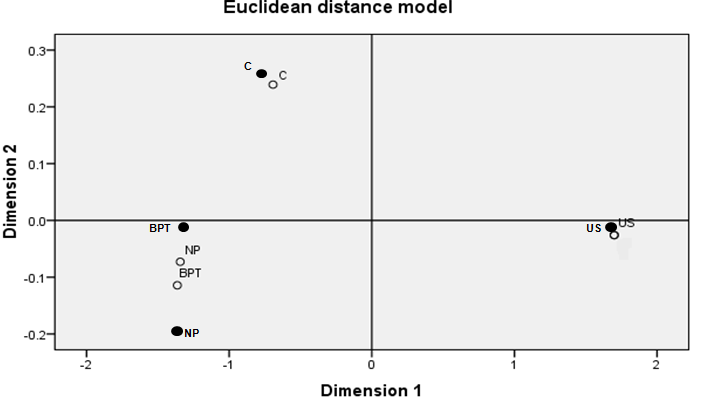


**S5 Fig**

**(A)**

**(B)**

**(C)**

Supplement: S5 Fig — Hollow circles = narG/nirK/nirS genes per g dry soil; solid circles = narG/nirK/nirS transcripts per g dry soil. (DOC) [file pone.0132770.s005.doc]
